# Partial or non-union after triple arthrodesis in children: does it really matter?

**DOI:** 10.1007/s11832-016-0730-z

**Published:** 2016-04-02

**Authors:** Eric D. Wicks, Melanie A. Morscher, Meadow Newton, Richard P. Steiner, Dennis S. Weiner

**Affiliations:** Northeast Ohio Medical University, Rootstown, OH 44272 USA; Akron Children’s Hospital, Akron, OH 44308 USA; Department of Statistics, University of Akron, Akron, OH 44325 USA; Department of Pediatric Orthopaedic Surgery, Akron Children’s Hospital, Akron, OH USA; Regional Skeletal Dysplasia Clinic, Akron Children’s Hospital, Akron, OH 44302 USA; Akron Children’s Hospital, 300 Locust Street, Ste. 250, Akron, OH 44302-1821 USA

**Keywords:** Triple arthrodesis, Radiographic non-union, Hindfoot deformity, Hindfoot fusion, Pseudoarthrosis, Subtalar joint, Calcaneocuboid joint, Talonavicular joint

## Abstract

**Purpose:**

Triple arthrodesis is a commonly performed salvage procedure to correct hindfoot deformity. Non-union is considered an undesirable radiographic outcome; however, the clinical ramifications of this are not as well defined. The purpose of this study was to determine the incidence of partial or complete radiographic non-union after triple arthrodesis in children and characterize the clinical consequences.

**Methods:**

An IRB-approved retrospective review of triple arthrodesis surgeries in patients less than 16 years of age performed by a single surgeon (DSW) identified 159 cases meeting the inclusion criteria. Plain radiographs were reviewed for bony fusion (defined as over 80 % radiographic bony union of the subtalar, calcaneocuboid, and talonavicular bones) and charts for clinical outcomes (pain, return to activity, and subsequent hindfoot surgeries). Statistics were used to compare the fused and unfused cases, with *p* < 0.05 considered to be significant.

**Results:**

Of the 159 cases included in the study, 9 % did not achieve at least 80 % plain film radiographic union. The fused and unfused groups had similar clinical outcomes. Only one patient required surgery for sequelae of symptoms arising from a pseudoarthrosis related to the triple arthrodesis. The fused and unfused groups were similar in terms of gender and pin removal time, but differed significantly in surgical age and underlying diagnosis.

**Conclusions:**

This is one of the largest case series of pediatric triple arthrodesis surgery presented in the literature. This study demonstrated that good clinical outcomes can be achieved despite the lack of radiographic union after triple arthrodesis surgery in children.

**Level of evidence:**

IV.

**Electronic supplementary material:**

The online version of this article (doi:10.1007/s11832-016-0730-z) contains supplementary material, which is available to authorized users.

## Introduction

Triple arthrodesis is a commonly performed salvage procedure to correct hindfoot deformity resulting from a myriad of diagnoses [1–14]. It is typically used to correct residual equines, equinovalgus, equinovarus, calcaneovalgus, or valgus deformities in adults or children [4, 5, 9, 14–16]. In triple arthrodesis surgery, the subtalar, calcaneocuboid, and talonavicular joints are fused to correct the hindfoot deformity. Good functional and often pain palliating outcomes have also been reported in the literature [1–24]. The surgery was first described by Hoke in 1921, but has been modified over the years, most notably by Ryerson, Lambrinudi, and Duncan [6, 7, 9, 18]. Most recently, an arthroscopic approach has been described in the literature [25–27].

One of the most commonly reported undesired outcomes of triple arthrodesis surgery is radiographic non-union and pseudoarthrosis of one or more joints of the hindfoot. The rate of non-union ranges from 3.8 to 23 % in children and adolescents and 0 to 46 % in adults [1, 2, 4, 5, 8, 10–14, 17, 19–24, 27]. The highest incidence of bony union failure occurs in the talonavicular joint, followed by the calcaneocuboid and, rarely, subtalar joints [1, 2, 4, 5, 8, 10–13, 17, 19–24, 27]. However, the correlation between radiographic non-union with pain or surgical failure with recurrent deformity has not been established [1, 4, 5, 8, 9, 11, 23]. While significant pain has been found in approximately 40 % of subjects with non-unions at any of the joints attempted to be fused [1, 4, 5, 8, 9, 23], it is unknown as to why some are painful and others are clinically silent [8]. In addition, Seitz and Carpenter were unable to establish a link between non-union and recurrent deformity [11].

Factors leading to incomplete union have been described in the literature and include the absence of internal fixation, poor bony contact, and early weight-bearing [8, 13, 28, 29]. Early weight-bearing seems to have the greatest effect on disruption of joint union [13, 28, 29] and the vertical shear forces from early weight-bearing may contribute to the high incidence of non-union in the talonavicular joint [1]. Other factors that have been implicated in incomplete union include age [9, 13], the underlying diagnosis, surgical technique for fixation [11, 13, 18], and presence of deep wound infection [5].

The purpose of this study was to determine the incidence of partial or complete radiographic non-union after triple arthrodesis surgery in children and characterize the clinical consequences. In addition, we attempted to determine if there are any statistically significant risk factors that may lead to this non-union.

## Materials and methods

An IRB-approved, retrospective review of all triple arthrodesis surgeries in patients less than 16 years of age performed by a single surgeon (DSW) at a single institution from June 1971 to August 2006 identified 244 triple arthodeses. Cases were included if there was radiographic evidence of union at any time prior to 1 year, or if radiographic follow-up was greater than 1 year, a time deemed sufficient for union to occur. Of the 244 cases identified, 85 were excluded for insufficient information available, leaving 159 cases in 111 patients (80 males and 31 females) in the study. Cases were excluded because radiographs were not available (73 cases) or because there was less than 1 year of radiographic follow-up and no evidence of union (12 cases). It is to be emphasized that plain radiographs were utilized to mirror a standard method of imaging follow-up of surgical triple arthrodesis (no CT or MRI).

**Fig. 1:**
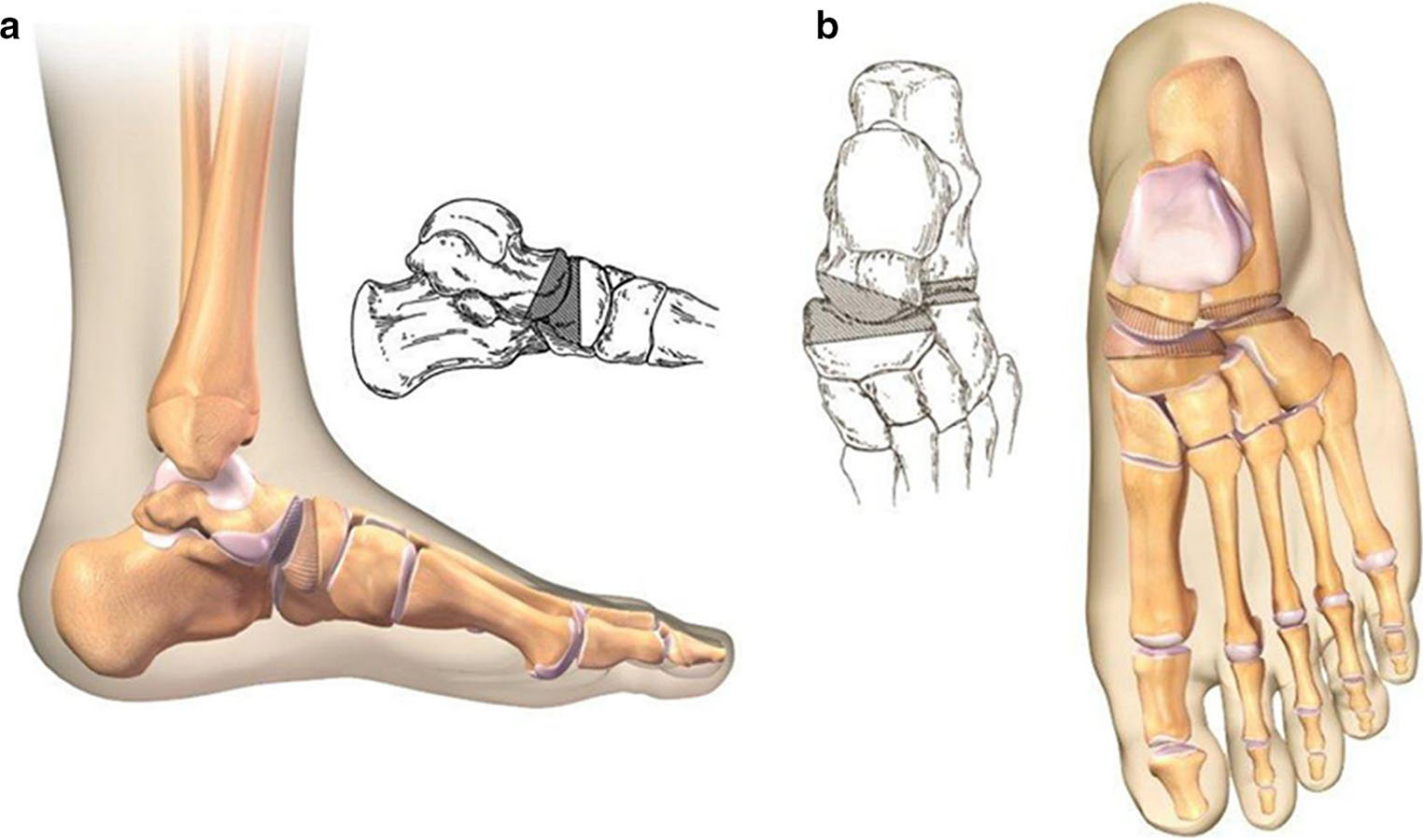
Representative medical illustrations of sections of bone removed during the procedure to provide conforming surfaces to be held in apposition for fusion of each joint from (**a**) medial and (**b**) anteroposterior views

**Fig. 2:**
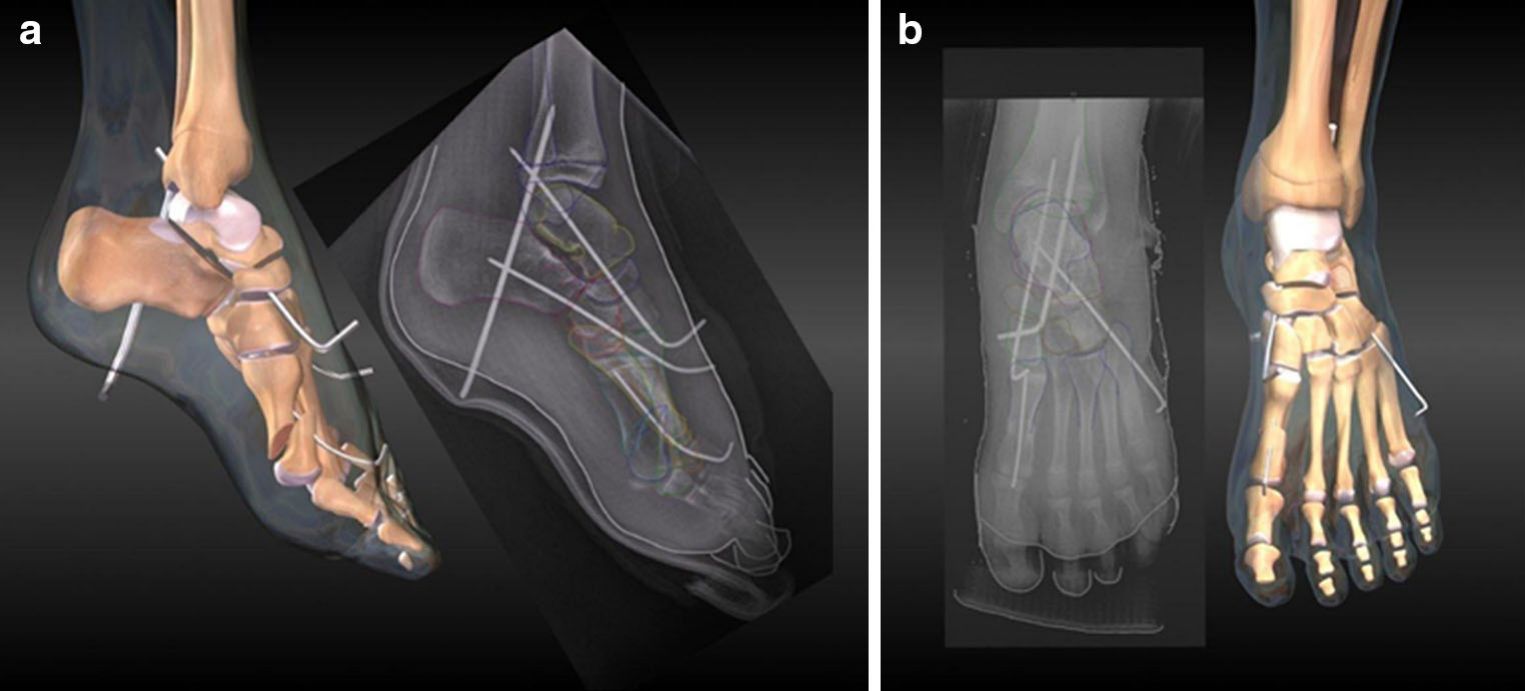
Representative images of the operative procedure including medical illustrations and radiographs of the completed triple arthrodesis with pin fixation of the subtalar, talonavicular, and calcaneocuboid joints from (**a**) medial and (**b**) anteroposterior views. In this case, another pin was placed in the first metatarsal because a metatarsal osteotomy was performed in conjunction with the triple arthrodesis

The senior author (DSW) consistently used the technique described below. The surgical approach was made through an oblique incision across the sinus tarsi to allow mobilization of soft tissue away from the subtalar joint. The short extensors were reflected from the os calcis, allowing exposure of the calcaneocuboid joint and access for further dissection along the neck and head of the talus. The joint capsule was elevated to visualize the talonavicular joint. Osteotomes and rongeurs were used to remove the promontory of the os calcis flush with the level of the subtalar joint (Fig. 1). The resected bone was cleared of cartilage, minced, and used for bone graft later in the procedure. The articular cartilage was denuded from the sustentaculum tali, cuboid, subtalar, and talonavicular joints. Two pins were inserted for stabilization across the subtalar joint and talonavicular joints. The subtalar pin was placed through the plantar aspect of the heel across the subtalar joint into the distal tibia, and the talonavicular pin was placed obliquely across the talonavicular joint. A third pin was utilized for fixation across the calcaneocuboid joint. Representative images of pin placement are shown in Fig. 2 and in an animation (Online Resource 1). Multiple sections of denuded bone fragments were packed across the subtalar, calcaneocuboid, and talonavicular joints. A well-padded short-leg cast was applied typically for 6 weeks when the pins were removed and a new short-leg cast applied. Weight-bearing was allowed when substantial radiographic union was evident (average 10–12 weeks).

**Fig. 3:**
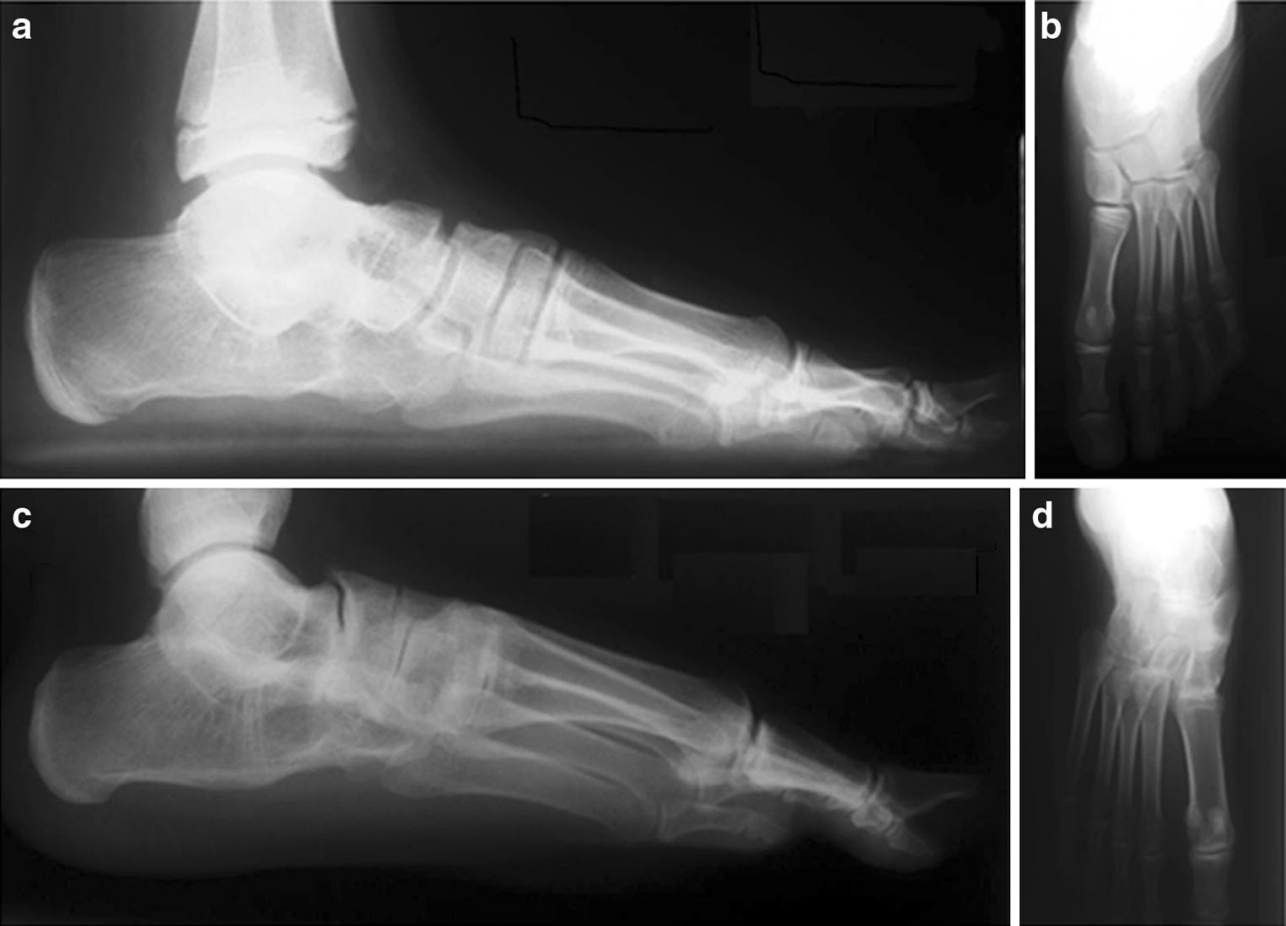
Radiographic examples of a complete union (**a**, **b**) and non-union (**c**, **d**) after a triple arthrodesis. The complete union radiographs were taken 1 year and 7 months after surgery in a male who had a triple arthrodesis surgery for a tarsal coalition at 13 years of age (**a**, **b**). The non-union radiographs were taken 2 years and 6 months after surgery in a male who had triple arthrodesis for a tarsal coalition and syndromic condition at 13 years of age (**c**, **d**)

Postoperatively, lateral and anteroposterior (AP) radiographs of the foot were reviewed by the authors for evidence of radiographic union of the subtalar, calcaneocuboid, and talonavicular bones. It is well recognized by the authors that plain radiographic examinations are inherently imperfect due to the plane of the hindfoot joints (particularly subtalar) being obliquely oriented relative to the incident beam of radiation. However, plain radiographic examinations are the standard used by orthopedic surgeons in initially determining fusion. For the purpose of this study, over 80 % radiographic bony union of the subtalar, calcaneocuboid, and talonavicular bones had to occur for the triple arthrodesis to be considered fused. The decision on percentage was purely arbitrary and the authors realize that any percentage of bony fusion probably represents a clinical immovable joint. Furthermore, variable non-radiologic union may represent fibrodesis and similar lack of mobility. All partial unions (less than 80 %) and non-unions of any of the three bones fused were classified as non-unions. Representative images of a complete and non-union are presented in Fig. 3.

All charts were reviewed for the following subjective and clinical outcomes: pain, return to previous activity, and subsequent hindfoot surgeries, including surgery for consequences of non-union or clinical symptoms. Additional demographic data were obtained from the charts. The average age at surgery was 11.4 years (range 7.0–15.9 years), the average clinical follow-up was 6.1 years (range 0.2–18.3 years), and the average radiographic follow-up was 4.1 years (range 0.2–16.4 years).

Statistics used to compare the patients experiencing complete union with those who did not included Chi-square tests for categorical variables and two-sample *t*-tests, Wilcoxon rank-sum tests, or median tests, as appropriate, for quantitative variables. Statistical significance was defined as *p* < 0.05.

## Results

Of the 159 cases included in the study, only 14 cases (8.8 %) did not achieve complete radiographic bony union. There were five cases of non-union and nine cases of partial union. Demographic information on these cases is presented in Table 1. The joints involved in the 14 cases that did not achieve complete radiographic union are presented in Table 2.

**Table 1:** Characteristics of the triple arthrodesis partial or non-union cases identified in this study

Pt^b^	Gdr	Diagnosis category	Side	Complications	Pain description	Active	Prev surg^b^	Subs surg^b^	Subs surg	SubTal X-ray union
2	M	CMT	R	None	^a^	Y	0	0		Y
4.1	M	CMT, tarsal coalition	R	Superficial skin necrosis	Chronic	^a^	0	0		Y
42	F	Clubfoot	R	None	Only with prolonged activity	Y	2	0		Y
66.1	M	CMT	R	None	^a^	Y	0	0		Y
82	M	Tarsal coalition, syndrome	L	None	Occasional	^a^	0	0		Y
8.1	M	CP	R	None	^a^	^a^	0	1		Y
8.2	M	CP	L	None	^a^	^a^	0	1		Y
12	M	Tarsal coalition	L	Wound infection	Occasional	Y	0	1	Triple revision	Y
14	M	CP	L	None	^a^	^a^	0	1	Akron dome	Y
21.1	M	Clubfoot	R	None	None	Y	1	0		Y
26.1	F	CP, hypotonic	R	None	^a^	^a^	0	0		Y
74	F	Clubfoot	R	None	None	^a^	4	0		70 %
79.1	M	Diplegia, TBI	R	None	^a^	Y	1	1	Soft tissue	Y
86.1	M	Tarsal coalition	L	None	None	Y	0	0		30 %

Pt^b^	Gdr	Cal-Cub X-ray union	Tal-Nav X-ray union	X-ray union category	Clinic FUP (years)	X-ray FUP (years)	Surg age (years)	Pins out (weeks)	Cast off (weeks)	FWB (weeks)
2	M	Y	Non-union	Non-union	8.1	6.7	9.6	5.6	9.6	20.4
4.1	M	Non-union	Y	Non-union	6.0	4.9	15.2	^a^	13.4	^a^
42	F	Non-union	Non-union	Non-union	1.7	1.5	13.6	^a^	12.9	29.4
66.1	M	Y	Non-union	Non-union	1.4	1.4	15.4	5.1	9.1	^a^
82	M	Y	Non-union	Non-union	2.6	2.6	14.0	6.3	9.3	^a^
8.1	M	Y	70 %	Partial union	4.2	1.1	11.6	7.4	13.4	17.3
8.2	M	Y	70 %	Partial union	4.2	1.1	11.6	7.4	13.4	17.3
12	M	Y	80 %	Partial union	2.4	2.0	15.8	5.9	10.9	^a^
14	M	50 %	Y	Partial union	2.6	2.1	11.1	^a^	10.4	16.7
21.1	M	30 %	Y	Partial union	9.1	9.1	10.1	5.4	8.4	^a^
26.1	F	Y	50 %	Partial union	7.4	1.6	11.4	6.4	11.4	^a^
74	F	Y	Y	Partial union	8.3	8.3	10.5	^a^	10.1	49.1
79.1	M	Y	80 %	Partial union	11.6	1.2	11.1	5.4	9.4	^a^
86.1	M	Y	Y	Partial union	1.0	1.0	14.0	7.1	11.1	^a^

*Cal-Cub* calcaneocuboid, *CMT* Charcot–Marie–Tooth disease, *CP* cerebral palsy, *F* female, *FUP* follow-up, *FWB* full weight-bearing, *Gdr* gender, *L* left, *M* male, *Prev* previous, *Pt* patient, *R* right, *Subs Surg* subsequent surgery, *SubTal* subtalar, *Tal-Nav* talonavicular, *TBI* traumatic brain injury, *Y* yes

^a^Not mentioned in chart

^b^Number

**Table 2:** Joints involved in the 14 cases that did not achieve complete radiographic union

Joint	Number of cases
Talonavicular	8 cases (5 partial non-unions; 3 non-unions)
Calcaneocuboid	3 cases (2 partial non-unions; 1 non-union)
Subtalar	2 cases (both partial non-unions)
Talonavicular and calcaneocuboid	1 case (both non-unions)

Compared to the complete union group, the non-union or partial union group had similar clinical and subjective outcomes (Table 3). In both groups, all patients returned to their previous or improved activities and approximately 85 % had no or only occasional pain with prolonged activities. Although many patients underwent other forefoot and midfoot surgical procedures, only one patient required surgery for sequelae of symptoms arising from a pseudoarthrosis of one or more of the joints of the triple arthrodesis. Revision surgeries in the complete union group were required only to achieve better anatomical alignment. Complications were minimal.

**Table 3:** Summary of clinical and subjective outcomes for the complete union and partial or non-union groups. Since this was a retrospective review, only outcomes mentioned in the chart are included in the percent calculation

Outcomes	Non-union group (*n* = 14)	Complete union group (*n* = 145)	*p*-Value
Pain			
No pain or occasional pain	86 %^a^	85 %^a^	0.959
Constant pain	14 %^a^	15 %^a^	
Return to previous activity	100 %^a^	100 %^a^	n/a
Subsequent triple revision surgery	1 case	8 cases	0.802
Complications			
Wound infection	7 %	7 %	0.997
Other	7 %	6 %	

*n/a* not applicable, *n* sample size

^a^Sample sizes for analyses were seven for the non-union group and 80 and 76 for the complete union group, for the pain and activity outcomes, respectively

The complete union and partial or non-union groups were statistically similar in terms of gender (71 vs. 79 % male), average pin removal time (5.8 vs. 6.2 weeks), and average clinical (6.2 vs. 5.0 years) and radiographic follow-up (4.2 vs. 3.2 years). Understandably, the average time in the cast was significantly longer in the partial or non-union group compared to the complete union group (10.9 vs. 10.0 weeks, *p* = 0.041), as was the median time to full weight-bearing (18.9 vs. 14.6 weeks, *p* = 0.009). The partial or non-union group compared to the complete union group was also significantly older (12.5 vs. 11.3 years, *p* = 0.049), had fewer previous surgeries (29 vs. 60 %, *p* = 0.026), and significantly different underlying diagnoses (Table 4).

**Table 4:** Underlying diagnoses in the complete union and partial or non-union groups

Diagnosis	Partial and non-union group (*n* = 14) (%)	Complete union group (*n* = 145) (%)	*p*-Value
Clubfoot	21	29	0.023
Charcot–Marie–Tooth disease	21	4	
Neurologic disorder^a^	37	41	
Tarsal coalition	21	10	
Other	0	16	

^a^Included cerebral palsy, diplegia, myelomeningocele, and traumatic brain injury

## Discussion

To the best of our knowledge, this is one of the largest case series of triple arthrodesis surgeries exclusively in children reported in the literature. Only 14 cases (8.8 %) of the 159 pediatric patients less than 16 years of age failed to achieve complete radiographic bony union. If one considers only the five complete radiographic non-unions, the rate of non-union in our series is 3.1 %. This is within the range of non-union rates reported in the literature for children and adolescents of 3.8 to 23 % [1, 2, 5, 11, 14, 20, 23], and most comparable to the non-union rates of 8.5 % (62 surgeries) and 9 % (66 surgeries) reported by Adelaar et al. and Seitz and Carpenter, respectively [1, 11]. In our study, the non-union rates for the talonavicular, calcaneocuboid, and subtalar joints (14 %) are also comparable to those reported in previous studies [1, 2, 4, 5, 8, 10, 11, 13, 17, 20, 22–24]. Eighty-five cases were excluded because radiographs could not be located (73 cases) or because there was less than 1 year of radiographic follow-up and no evidence of union (12 cases).

The presence of a non-union would empirically suggest that the surgery was unsuccessful and that further discomfort would be presumed to occur. Pain associated with non-union has been described with great variability, from no pain [4, 5] to significant pain in patients with incomplete union [23]. Patterson et al. were unable to explain why some incomplete unions were painful and others were not pain generating [8]. In their study, talonavicular pseudoarthrosis accounted for 89 % of all incomplete unions, but only one-fifth presented with pain. Our findings demonstrated no significant difference between the union and non-union groups with respect to pain. Eighty-six percent of the patients in the non-union group were without pain or only experienced occasional pain, compared to 85 % in the complete union group. In addition, all patients in both groups returned to their previous functional levels or improved levels.

Ryerson predicted that revision surgery would not be needed in pseudoarthrosis [9]. Others did revise some, but not all, painful incomplete unions [8, 11, 23, 24]. Our findings are similar in that only one patient required surgery for sequelae of symptoms arising from a pseudoarthrosis; however, there was no mention of pain in the medical record.

The non-union and complete union groups were similar in many respects. No significant differences were found between the two groups in respect to pin removal time and gender (*p* > 0.05). However, the non-union group, when compared to the complete union group, tended to remain non-weight-bearing longer, be significantly older, with fewer previous surgeries, and different underlying diagnosis. Many of these factors were suggested in prior studies [8, 11, 13, 18, 28, 29]. It would seem logical to speculate that fibrous union (pseudoarthroses) was sufficient to restrict motion in the non-union group and, thereby, preclude discomfort. It must be restated that any bony union visible on plain radiographs is likely connected with diminished or absent motion at that particular joint.

## Conclusion

This study represents one of the largest case series of pediatric triple arthrodesis surgery presented in the literature. This study demonstrated that good clinical outcomes can be achieved despite the lack of radiographic union after triple arthrodesis surgery in children.

## Electronic supplementary material

Below is the link to the electronic supplementary material.

Representative three-dimensional animation of the completed triple arthrodesis with pin fixation of the subtalar, talonavicular, and calcaneocuboid joints. In this case, another pin was placed in the first metatarsal because a metatarsal osteotomy was performed in conjunction with the triple arthrodesis (WMV 2348 kb)
